# Economic evaluation of the one-hour rule-out and rule-in algorithm for acute myocardial infarction using the high-sensitivity cardiac troponin T assay in the emergency department

**DOI:** 10.1371/journal.pone.0187662

**Published:** 2017-11-09

**Authors:** Apoorva Ambavane, Bertil Lindahl, Evangelos Giannitis, Julie Roiz, Joan Mendivil, Lutz Frankenstein, Richard Body, Michael Christ, Roland Bingisser, Aitor Alquezar, Christian Mueller, for the TRAPID-AMI investigators

**Affiliations:** 1 Modeling and Simulation, Evidera, London, United Kingdom; 2 Department of Medical Sciences, Uppsala University and Uppsala Clinical Research Center, Uppsala, Sweden; 3 Medizinische Klinik III, University Heidelberg, Heidelberg, Germany; 4 Previous employment: Market Access, Roche Diagnostics International Ltd., Rotkreuz, Switzerland; 5 Department of Cardiology, Angiology, Pulmonology, University Hospital of Heidelberg, Heidelberg, Germany; 6 Emergency Department, Central Manchester University Hospitals NHS Foundation Trust, Manchester, United Kingdom; 7 Department of Emergency and Critical Care Medicine, Paracelsus Medical University, Nuremberg General Hospital, Nuremberg, Germany; 8 Emergency Department, University of Basel, University Hospital, Basel, Switzerland; 9 Servei de Urgencies. Hospital de Sant Pau, Barcelona, Spain; 10 Department of Cardiology and Cardiovascular Research Institute Basel, University Hospital Basel, Basel, Switzerland; Universitatsklinikum Hamburg-Eppendorf, GERMANY

## Abstract

**Background:**

The 1-hour (h) algorithm triages patients presenting with suspected acute myocardial infarction (AMI) to the emergency department (ED) towards “rule-out,” “rule-in,” or “observation,” depending on baseline and 1-h levels of high-sensitivity cardiac troponin (hs-cTn). The economic consequences of applying the accelerated 1-h algorithm are unknown.

**Methods and findings:**

We performed a post-hoc economic analysis in a large, diagnostic, multicenter study of hs-cTnT using central adjudication of the final diagnosis by two independent cardiologists. Length of stay (LoS), resource utilization (RU), and predicted diagnostic accuracy of the 1-h algorithm compared to standard of care (SoC) in the ED were estimated. The ED LoS, RU, and accuracy of the 1-h algorithm was compared to that achieved by the SoC at ED discharge. Expert opinion was sought to characterize clinical implementation of the 1-h algorithm, which required blood draws at ED presentation and 1h, after which “rule-in” patients were transferred for coronary angiography, “rule-out” patients underwent outpatient stress testing, and “observation” patients received SoC. Unit costs were for the United Kingdom, Switzerland, and Germany. The sensitivity and specificity for the 1-h algorithm were 87% and 96%, respectively, compared to 69% and 98% for SoC. The mean ED LoS for the 1-h algorithm was 4.3h—it was 6.5h for SoC, which is a reduction of 33%. The 1-h algorithm was associated with reductions in RU, driven largely by the shorter LoS in the ED for patients with a diagnosis other than AMI. The estimated total costs per patient were £2,480 for the 1-h algorithm compared to £4,561 for SoC, a reduction of up to 46%.

**Conclusions:**

The analysis shows that the use of 1-h algorithm is associated with reduction in overall AMI diagnostic costs, provided it is carefully implemented in clinical practice. These results need to be prospectively validated in the future.

## Introduction

A total of 5% to 10% of all visits to the emergency department (ED) are related to patients presenting with chest pain or other symptoms suggestive of acute coronary syndrome.[1–3] The accurate diagnosis of acute myocardial infarction (AMI) in such patients is often difficult and time-consuming, and delays in diagnosis may increase the risk of complications and mortality.[4, 5] Until recently, the diagnostic and triage tools used by physicians were clinical symptoms, patient history, the 12-lead electrocardiogram (ECG) and standard cardiac troponin (cTn) assays.[4, 6–8]

Standard cTn assays require serial sampling for 6 to 12 hours because the increase in troponin level can be delayed until 3 to 4 hours following the onset of AMI.[6, 8, 9] This limits how quickly patients with suspected AMI can be either “ruled-in” or “ruled-out”, meaning that, although only 25% of them truly have the condition, 80% end up having a prolonged stay in the ED, or being admitted to the hospital for clinical observation.[10] Thus, early reliable ruling-in or ruling-out of AMI can ensure timely receipt of evidence-based therapies for the patient’s underlying condition and avoid crowding of the ED.[4, 5, 11] Accordingly, for faster diagnosis of AMI,[12–14] recently published studies and treatment guidelines recommend the use of high-sensitivity cardiac troponin assays (hs-cTn), which enable the detection of lower limits of cTn concentration that were not reliably identified detected by the standard assays.[15]

The optimal use of hs-cTnT in clinical practice has been studied in a prospective, diagnostic, multicenter study (Advantageous Predictors of Acute Coronary Syndrome Evaluation Study—APACE), which included 872 patients who presented with acute chest pain to the ED.[16] This study presented a 1-hour (h) algorithm that categorized patients as “rule-out”, “rule-in”, or “observation”, depending on the baseline and absolute changes in 1-h levels of cTnT using hs-cTn.[16] The use of the algorithm was found to be have high sensitivity and specificity and prospectively validated in three prospective, diagnostic, studies, including TRAPID-AMI, which included 1,282 patients presenting with acute chest pain to the ED (The High Sensitivity Cardiac Troponin T Assay for Rapid Rule-out of Acute Myocardial Infarction—TRAPID-AMI).[16–18]

Such evidence of the reliability of a 1-h algorithm raises the possibility that its use could help to avoid serial blood sampling and prolonged monitoring of patients presenting with acute chest pain to the ED,[16, 17] and to ensure accurate and faster diagnosis to ensure that such individuals have quicker access to treatments.[15] This rationale is supported by previously published studies that assessed accelerated diagnostic protocols with high-sensitivity troponin assays (hs-cTnT or hs-cTnI) compared with standard troponin testing—these studies reported cost savings,[19–21] reduction in length of stay (LoS),[19, 20] and improvement in life-years and quality-adjusted life-years.[22, 23] Although the clinical performance of a 1-h algorithm was well validated, its implications on ED length of stay (LoS), resource utilization (RU), and economic consequences have not been studied. So in order to better understand the economic and clinical outcomes of implementing a 1-h algorithm in clinical practice, we conducted a study to estimate the diagnostic accuracy, LoS, RU, and cost consequences of the 1-h algorithm compared to standard of care (SoC) in the ED in the TRAPID-AMI study.

## Methods

### Dataset for the analysis

**Fig 1:**
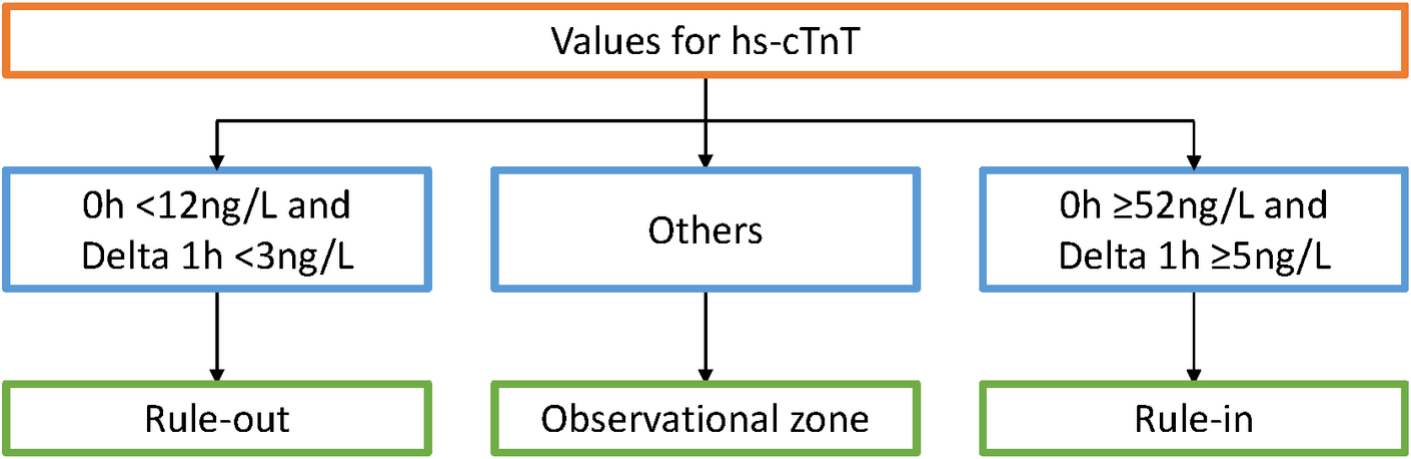
One-hour rule-out and rule-in Algorithm [16, 17].

The TRAPID-AMI[17] study enrolled patients who presented to the ED with acute chest pain (onset of chest pain or discomfort within the previous 6 hours; details of inclusion and exclusion criteria are presented in Mueller et al, 2016).[17] Blood samples were collected within 45 minutes of presentation to the ED and also 1 hour ± 30 mins after that.[17] The samples were assessed using the hs-cTnT and sensitive cardiac troponin I (s-cTnI-ultra) assay at a central core laboratory.[17] The 1-h algorithm classified patients as “rule-out,” “rule-in,” and “observation” based on the measurements of hs-cTnT levels at baseline and absolute changes at 1h (Fig 1).[16, 17] The ED physicians were blinded to the results of the 1-h algorithm, including results of the blood tests for hs-cTnT and s-cTnI-ultra.[17] Patients were diagnosed and triaged as per standard clinical practice (defined as SoC in this analysis). The study recorded hospital center, patient characteristics, clinical history, laboratory and imaging tests, procedures conducted, length of stay (LoS), and diagnosis at discharge (ED physician diagnosis).[9]

Two independent cardiologists who were part of a clinical endpoint committee (CEC) adjudicated on each patient’s record to determine the final diagnosis.[17] This determination was based on a comprehensive review of medical records for the patient from ED presentation to 30-day follow-up, physical examination findings, and s-cTnI-ultra and local cTn levels obtained before the first or after the last blood drawn for the study, and, when available levels of the following markers: serum creatinine, cystatin C, free hemoglobin, and NT-proBNP. Details of the following diagnostic investigations were also reviewed: cardiac stress test; ECG; echocardiography; radiologic imaging; and lesion severity and morphology on coronary angiography [17]

Using the TRAPID-AMI study dataset, the diagnostic accuracy, ED LoS, and RU were estimated for the SoC and 1-h algorithm.[9]

### Diagnostic accuracy

The current analysis defined SoC as the diagnostic and triaging pattern adopted by ED physicians in the TRAPID-AMI study. The diagnostic accuracy of SoC was estimated by calculating the sensitivity, specificity, NPV and PPV of AMI diagnosis at the time of ED discharge compared to the CEC adjudication. The accuracy of the “rule-out” classification of the 1-h algorithm was estimated by calculating the specificity and NPV, and the accuracy of “rule-in” was estimated by calculating the sensitivity and PPV. The patients in the “observation” group were assumed to be diagnosed in patterns that mirrored those associated with SoC; and hence the diagnostic accuracy of this classification was assumed to be similar to that of SoC. The diagnostic accuracy of the 1-h algorithm was calculated as the weighted average of estimates for “rule-out,” “rule-in,” and “observation” category. The diagnostic and treatment patterns for AMI differed by country of interest and subsequently, individual study site (specifically, Sydney, Brussels, Basel, Heidelberg, Nuremberg, Barcelona, Manchester, Milan, Padova, Stockholm, Baltimore, and Detroit); and therefore diagnostic accuracy was estimated for all TRAPID-AMI sites to understand the variation from the overall population.[9] Using the sensitivity estimates for 1-h algorithm and SoC, the proportion of patients with each of the following types of diagnoses were estimated: true-positive (sensitivity); false-negative (1 –sensitivity); true-negative (specificity); and false-positive (1 –specificity).

### ED length of stay

The ED LoS for SoC was calculated as the difference between the time of discharge from the ED and the time of presentation to the ED, as recorded in the TRAPID-AMI study.[9] Four data points representing outliers that were above the 90^th^ centile (65 hours) and which ranged between 69 and 101 hours were excluded when deriving the estimates for the ED LoS analysis,[8] based on clinical opinion that these observations were not plausible in routine clinical practice. Predictive equations were developed for ED LoS for patients with AMI diagnosis and those without this diagnosis (non-AMI) as defined by the ED physician. Parametric distributions, including Weibull, log-normal, log-logistic, exponential, generalized gamma, and Gompertz, were fitted to the ED LoS. Log-normal distribution was considered to provide the best fit to the observed ED LoS data based on standard goodness-of-fit measures (Akaike Information Criteria and Bayesian Information Criteria), log-cumulative hazard plots, visual inspection, and comparison of median ED LoS estimates of observed and fitted data.[8]

A univariate model was fitted to understand the impact of baseline patient characteristics on the ED LoS for patients with AMI and non-AMI diagnosis. The patient characteristics evaluated were based on clinical opinion and included age (> 75 years, > 65- < = 75 years, vs. < = 65 years); gender; time since onset of chest pain (continuous variable); time since peak of chest pain (continuous variable); dyspnea (presence vs. absence); chest pain intensity (1–10 vs. 0); blood pressure (above range, below range vs. normal range as per American Heart Association classification); heart rate (above range, below range vs. normal range as per American Heart Association classification); and history of congestive heart failure (yes, unknown vs. no). Furthermore, due to a variation in clinical practice across study sites, the impact of each study center on the ED LoS was assessed in the univariate analysis.

The covariates that showed significance (alpha level 10%) to ED LoS predictions for patients diagnosed with AMI were included in a multivariate regression model to derive predictive equations of ED LoS for patients with AMI and non-AMI diagnosis.

**Fig 2:**
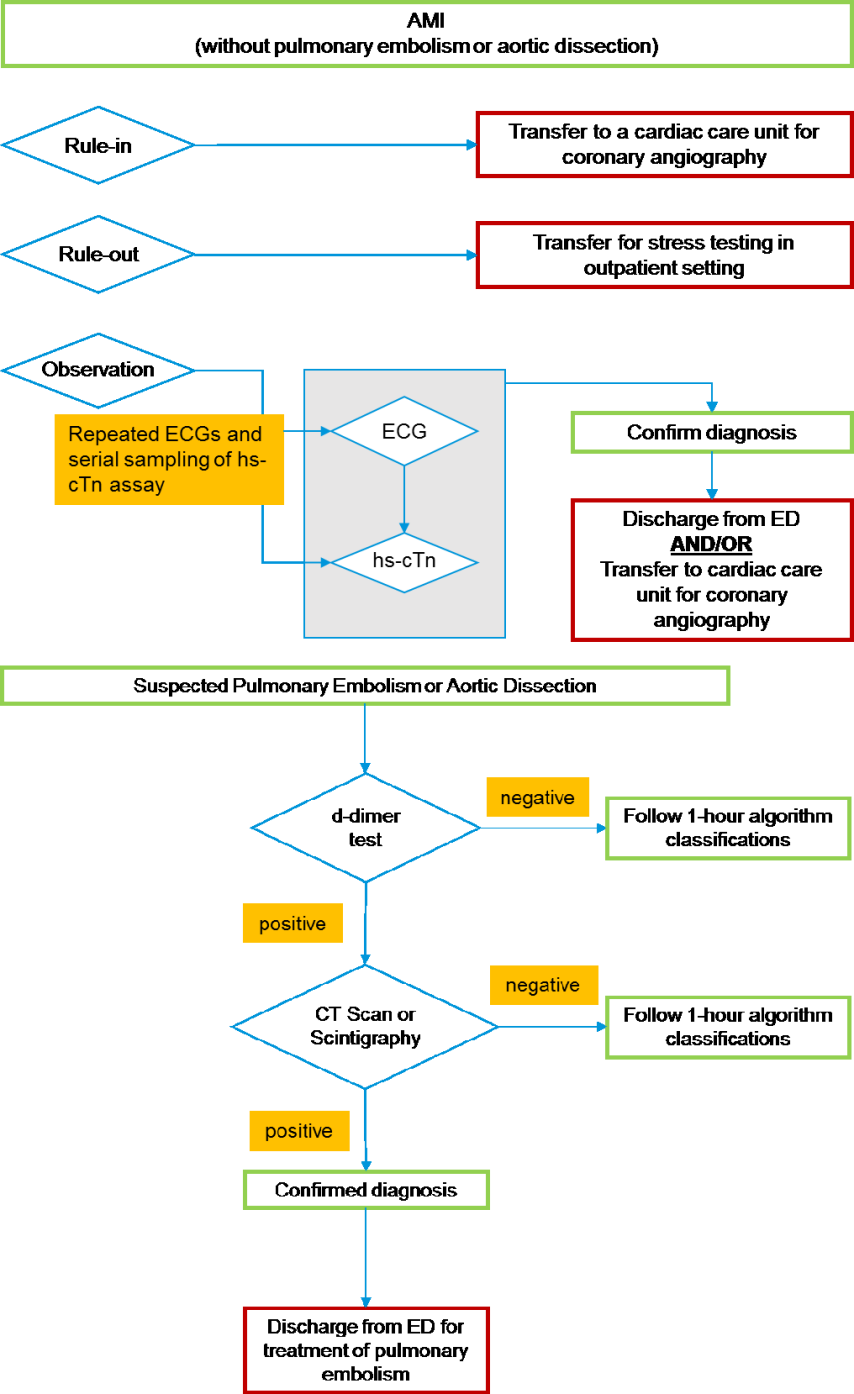
Clinical implementation of one-hour rule-out and rule-in algorithm. Abbreviations: AMI, acute myocardial infarction; ECG, electrocardiogram; ED, emergency department; hs-cTn, high-sensitivity cardiac troponin assays.

Since the ED physicians were blinded to the results of the 1-h algorithm, its practical implementation and impact on ED LoS were not directly recorded in the TRAPID-AMI study.[9, 17] Therefore, clinical opinion was elicited to understand how the 1-h algorithm would be implemented in practice. The clinicians suggested that patients categorized as “rule-in” would be discharged from the ED and transferred to the cardiac care unit of a hospital for coronary angiography; patients in the “rule-out” category would be discharged from the ED and receive an outpatient stress test; and those in the “observation” category would stay in the ED and undergo serial blood sampling and ECGs until a diagnosis could be confirmed (Fig 2).

**Fig 3:**
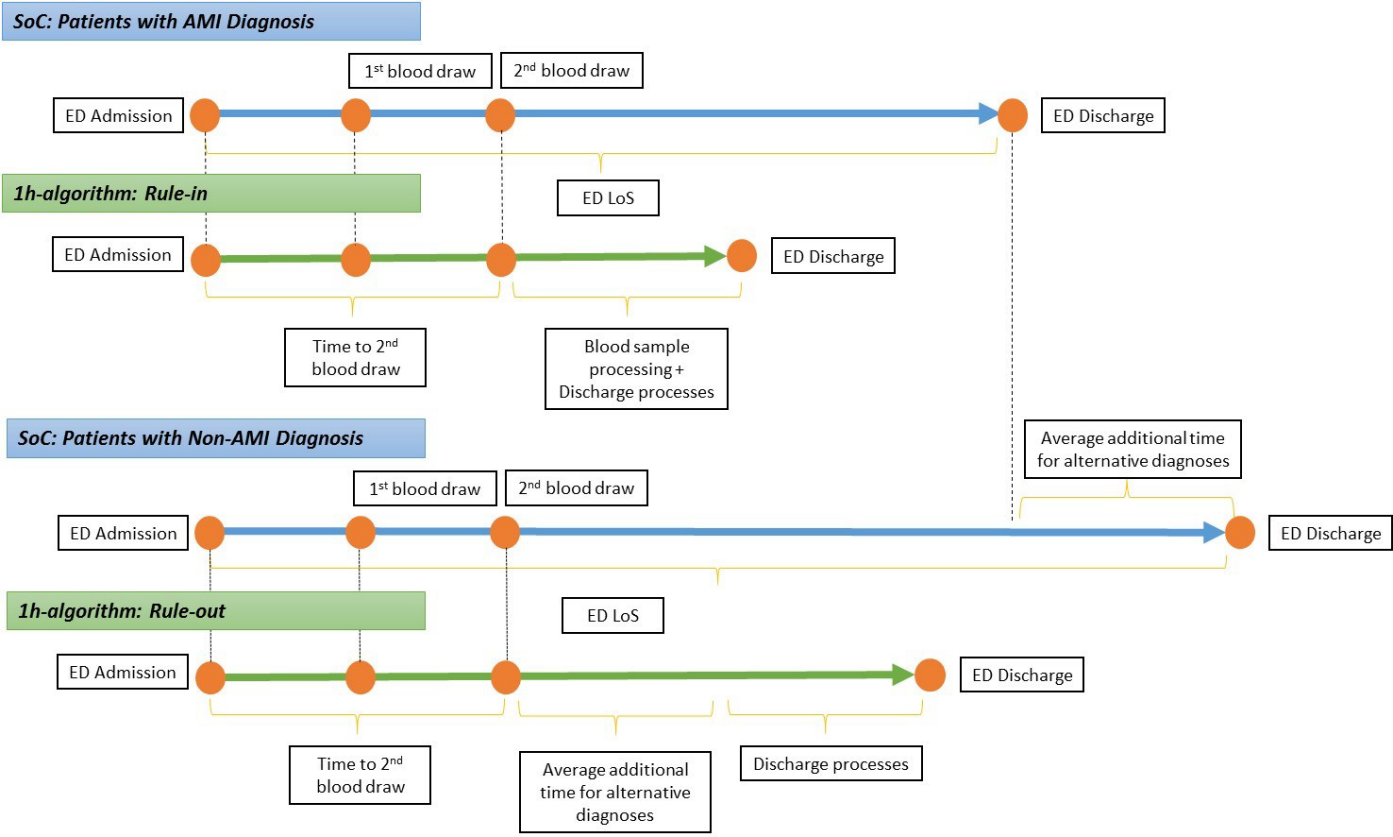
One-hour algorithm LoS estimation. Abbreviations: AMI, acute myocardial infarction; ED, emergency department; LoS, length of stay; SoC, standard of care.

Based on the expected implementation of the 1-h algorithm in clinical practice, patients in the “rule-in” and “rule-out” categories would have two blood draws in the ED. The time of second blood draw receipt was estimated from the TRAPID-AMI study.[8] Clinicians suggested that an average of 1 hour was required for analysis of blood samples and to implement the discharge protocol from the ED. Therefore, the ED LoS was estimated as the time from second blood draw to discharge from the ED for the “rule-in” and “rule-out” patients (Fig 3). Furthermore, it was expected that the “rule-out” patients may require additional time for confirming alternative diagnosis that is conducted as per SoC patterns. This time was estimated, using the SoC data from the TRAPID-AMI study, as the difference in the ED LoS between non-AMI and AMI patients (Fig 3).

### Resource use

The analysis evaluated RU patterns associated with SoC in the ED. Specifically, it reported the proportion of patients undergoing tests (serial blood draws, serial ECGs, computed tomography (CT) scan, magnetic resonance imaging (MRI) scan, echocardiography) and procedures (invasive angiography, coronary artery bypass graft (CABG) surgery, percutaneous transluminal coronary angiography (PTCA), and coronary stent placement), and the associated time since admission to the ED. Continuous variables were presented as mean and standard errors and categorical variables as percentage and numbers. RU and associated times were stratified by the presence or absence of an AMI diagnosis, as per the ED physicians’ adjudication. Furthermore, the impact of study site on RU was assessed. RU was classified over time: before admission to the ED, defined as the time between the receipt of resource and ED admission time; during ED stay, defined as the time between ED admission and ED discharge; and post ED discharge, defined as the time between ED discharge and receipt of procedure.

Since the ED physicians were blinded to the results of the 1-h algorithm, RU with the algorithm was based on clinical opinion on the implementation of the 1-h algorithm in routine practice (Fig 3). The analysis assumed that RU before admission to the ED for 1-h algorithm would be similar to SoC. Also RU for “rule-in” and “rule-out” patients would be similar to SoC until the occurrence of the second blood draw during ED stay. Other tests observed in SoC after the second blood draw were not considered in the analysis, based on clinical opinion on how the 1-h algorithm would be implemented. After discharge from the ED, “rule-in” patients were expected to have procedures similar to those used in SoC patients: invasive angiography, CABG, PTCA, and stent for AMI treatment. The 1-h algorithm was developed only for the diagnosis of patients; hence, the treatment of patients was expected to be similar to the SoC. “Rule-out” patients underwent outpatient stress tests after discharge from the ED. The patients in the “observation” category were assumed to have RU similar to SoC.

Patients with a false-positive or false-negative diagnosis of AMI were assumed to require inpatient treatment for their true underlying condition, based on clinical opinion. The true diagnosis of patients was based on CEC adjudication (Table C in S1 Appendix).

### Costs

Unit costs for RU and inpatient treatment of AMI and alternative diagnoses were estimated using publicly available costs from the perspective of payers in the United Kingdom (UK),[24, 25] Germany,[26, 27] and Switzerland.[28–31] The costs are reported in 2016 Great British Pounds (£), 2016 Euros (€), and 2016 Swiss Francs (CHF).

### Sensitivity analysis

One-way sensitivity analysis was conducted to understand the impact of various parameters on the economic analysis results. The sensitivity, specificity, negative predictive value (NPV), positive predictive value (PPV), coefficients and means in the predictive equations, proportion of patients using resources, and the time of RU were varied +/- standard error (SE) in the analysis. These SEs were obtained from the TRAPID-AMI study data. The costs were varied +/-20% of the mean.

## Results

### Diagnostic accuracy

Table 1 presents the diagnostic accuracy, using the sensitivity, specificity, NPV and PPV measures, for the “rule-in,” “rule-out,” and “observation” categorization of the 1-h algorithm and SoC. When judged against the CEC adjudication, the sensitivity for “rule-in” categorization of the 1-h algorithm was higher than that for SoC (95% vs. 69%, respectively), while the specificity for “rule-out” categorization of the algorithm was similar to that for SoC (95% vs. 98%, respectively). Overall, the 1-h algorithm, including “rule-in,” “rule-out,” and “observation” zone, was associated with higher sensitivity and similar specificity compared to SoC.

**Table 1:** Sensitivity, specificity, negative predictive value and positive predictive value for 1-h algorithm and SoC.

	No. of Patients	Sensitivity (95% CI)	Specificity (95% CI)	PPV (95% CI)	NPV (95% CI)
**1-h algorithm (rule-in and rule–out) vs. CEC adjudication**	997	95% (91%-98%)	95% (93%-96%)	77% (70%-83%)	99% (98%-99.5%)
Observation zone* **vs. CEC adjudication**	285	69% (62%-75%)	98% (97%-99%)	90% (84%-94%)	94% (92%-95%)
1-h algorithm (rule-in/out + observation)** **vs. CEC adjudication**	1282	87%	96%	80%	97%
**SoC vs. CEC adjudication**	1282	69% (62%-75%)	98% (97%-99%)	90% (84%-94%)	94% (92%-95%)

Abbreviations: CI, confidence interval; NPV, negative predictive value; PPV, positive predictive value; SoC, standard of care

*Assumed same as SoC

**Weighted average of rule-in/ rule-out and observation

### Length of stay

#### Standard of care

The multivariate regression model for LoS of patients with AMI and non-AMI diagnosis for SoC in the ED is reported in Table 2. The covariates included were study site, time since peak of chest pain, dyspnea, and congestive heart failure. For patients with a non-AMI diagnosis, ED LoS predictors were study site, age, time since peak of chest pain, dyspnea, chest pain intensity, heart rate, and history of congestive heart failure. The mean ED LoS for patients with AMI diagnosis was estimated to be 5.3 hours and for non-AMI diagnosis to be 6.6 hours (weighted mean: 6.5 hours). The ED LoS varied across study sites. Patients with AMI diagnosis in the ED had shorter LoS compared to those with alternative diagnoses (Fig. A in S1 Appendix), and this was observed across all study sites except for that in Heidelberg. There was a large variation in mean ED LoS across sites; the mean ED LoS was longer (11.6–16.8 hours) for specific study sites such as Barcelona, Milan, and Sydney, while shorter mean ED LoS was found for study sites, such as Nuremberg, Padova, and Stockholm sites (2.2–3.1 hours; Fig. A in S1 Appendix).

**Table 2:** Multivariate regression model for LoS of patients with AMI and non-AMI diagnosis: SoC.

		AMI				Non-AMI			
Parameter		Co-efficient	95% CI		P-value > Chi-square	Co-efficient	95% CI		P-value > Chi-square
**Intercept**		1.7949	0.1447	3.4451	0.033	2.0077	1.728	2.2874	< .0001
**Investigator site**	Sydney	0.5209	-1.1899	2.2317	0.5506	0.7926	0.5773	1.0078	< .0001
**Investigator site**	Brussels	-0.0589	-2.1545	2.0366	0.956	0.2363	-0.0284	0.501	0.0801
**Investigator site**	Basel	0.5366	-1.1544	2.2276	0.534	0.197	-0.0581	0.4521	0.1302
**Investigator site**	Heidelberg	0.6571	-0.9985	2.3127	0.4366	0.0031	-0.2694	0.2755	0.9824
**Investigator site**	Nuremberg	-0.1371	-1.9488	1.6746	0.8821	-0.6486	-1.0535	-0.2437	0.0017
**Investigator site**	Barcelona	1.2668	-0.3821	2.9157	0.1321	1.0026	0.8009	1.2043	< .0001
**Investigator site**	Manchester	0.3381	-1.4173	2.0935	0.7058	-0.2656	-0.6174	0.0862	0.1389
**Investigator site**	Milan	1.0565	-0.6068	2.7198	0.2131	0.6906	0.482	0.8992	< .0001
**Investigator site**	Padova	-0.5153	-3.6645	2.634	0.7485	-0.0456	-0.5485	0.4573	0.8589
**Investigator site**	Stockholm	-0.4207	-2.3334	1.492	0.6664	-0.6901	-1.1847	-0.1955	0.0062
**Investigator site**	Baltimore	0.0726	-1.7421	1.8874	0.9375	0.0806	-0.183	0.3443	0.5489
**Investigator site**	Detroit	0				0			
**Age category (years)**	>65 to 75					0.0899	-0.0208	0.2007	0.1115
**Age category (years)**	>75					0.1646	0.0573	0.2719	0.0027
**Age category (years)**	≤65					0			
**Peak time of chest pain**	Continuous	-0.0906	-0.2048	0.0237	0.1203	-0.0277	-0.0626	0.0073	0.1209
**Dyspnea**	Yes	-0.2858	-0.5512	-0.0204	0.0348	0.1532	0.065	0.2413	0.0007
**Dyspnea**	No	0				0			
**Intensity of chest pain**	1–10					-0.3053	-0.5043	-0.1063	0.0026
**Intensity of chest pain**	0					0			
**Heart rate**	Above normal range					0.0229	-0.1118	0.1577	0.739
**Heart rate**	Below normal range					0.1253	-0.0011	0.2518	0.0521
**Heart rate**	Normal range					0			
**Congestive heart failure**	Unknown	-1.4077	-5.7665	2.9512	0.5268	-0.3637	-2.0954	1.3679	0.6806
**Congestive heart failure**	Yes	-0.3443	-1.0141	0.3256	0.3138	0.0024	-0.1781	0.1829	0.9792
**Congestive heart failure**	No	0				0			
**Scale**		6.3127	5.6456	7.0586		6.1801	5.9205	6.4511	

Abbreviations: AMI, acute myocardial infarction; CI, confidence interval; SoC, standard of care

#### One-hour algorithm

The time from admission to second blood draw is summarized by study sites in Fig. B in S1 Appendix. The observed mean time to second blood draw was 1.7 hours (SE: 0.03 hours) and was similar across study sites. Table 3 presents the estimated mean ED LoS for the “rule-in” and “rule-out” categorization of the 1-h algorithm. The mean ED LoS for patients with “rule-in” and “rule-out” categorization was estimated to be 2.7 hours and 4.0 hours, respectively (Fig. C in S1 Appendix). The estimated mean ED LoS varied across sites; study sites such as Barcelona, Brussels, Milan, and Sydney were found to have longer ED LoS compared to those at Nuremberg, Heidelberg, Manchester, and Stockholm (mean ranges: 6.1 to 7.9 hours vs. 2.3 to 3.1 hours, respectively; Fig. D in S1 Appendix).

**Table 3:** Mean ED LoS for 1-h algorithm.

	Rule-in	Rule-out	Observation–AMI	Observation–non-AMI
Number of patients	184	813	48	287
Mean time from admission to 2d blood test	1.7 hours	1.7 hours	—	—
Mean additional time for alternative diagnosis	—	1.3 hours*	—	—
Mean time to discharge	1.0 hoursᵻ	1.0 hoursᵻ	—	—
**Mean ED LoS**	**2.7 hours**	**4.0 hours**	5.3 hours**	6.6 hours**
Mean ED LoS 1-h algorithm***	**4.3 hours**			

Abbreviations: AMI, acute myocardial infarction; ED LoS, emergency department length of stay

*Difference in LoS for alternative diagnosis compared to AMI diagnosis with SoC in the ED

ᵻAssumption based on clinical opinion

**Assumed to be same as SoC

***Weighted average of rule-in/ rule-out and observation

### Resource use

#### Standard of care

RU in the ED for patients with AMI diagnosis and non-AMI diagnosis is presented in Table A in S1 Appendix. The proportion of patients receiving the resources of interest before admission to the ED, during their ED stay, and post-ED discharged is detailed in the S1 Appendix. Furthermore, the unit costs from the perspective of national payers in the UK, Germany, and Switzerland are presented. Of note, RU varied across study sites, with respect to the number of serial blood draws and ECGs, and receipt of CT and MRI scans, echocardiography, and various procedures (refer to Fig. F1-F3 in S1 Appendix).

#### One-hour algorithm

Table B in S1 Appendix summarizes the estimated RU with the 1-h algorithm for patients categorized as rule-in and rule-out (as detailed in the *Methods* section). The “rule-in” and “rule-out” patients achieve reduced RU due to their quicker diagnosis, and shorter LoS. Specific resources avoided include blood draws, ECGs, CT scans, MRI scans, and procedures (invasive angiography, PTCA, stent, and CABG) performed in the ED. Based on clinical opinion, “rule-in” patients were assumed to have similar procedure requirements as the patients with AMI diagnosis in the SoC arm following discharge from the ED. By comparison, “rule-out” patients were assumed to require outpatient stress tests after discharge from the ED; and patients classified as observation were assumed to require similar RU as SoC.

### Cost-consequence analysis

Table 4 summarizes the proportion of patients with true-positive, false-negative, true-negative, and false-positive diagnoses using the sensitivity and specificity estimates for 1-h algorithm, including “rule-in,” “rule-out,” and “observation” patients, and SoC, as well as the LoS associated with 1-h algorithm and SoC. The 1-h algorithm was associated with a reduction in ED LoS of 2.1 hours (33% reduction) compared to SoC. The time saved in the ED varies by study site, with a higher reduction in ED LoS being found with respect to the Barcelona, Milan, Sydney, and Heidelberg sites (mean time saved range: 3.5 to 7.6 hours), and Padova, Stockholm, Baltimore, and Detroit showing lower reduction in ED LoS (mean time saved range: -0.2 to 1.0 hours; Fig. H in S1 Appendix).

**Table 4:** Diagnostic accuracy and mean ED LoS for 1-h algorithm compared to SoC.

**1-h Algorithm**				
	**True-Positive**	**False-Negative**	**True-Negative**	**False-Positive**
% patients: “rule-in”/”rule-out”	12.60%	0.62%	61.35%	3.20%
% patients: observation zone	2.61%	1.17%	18.16%	0.29%
Mean LoS	2.7 hours	4.0 hours	4.0 hours	2.7 hours
**Mean ED LoS: 1-h algorithm**	**4.34 hours**			
**SoC**				
	**True-Positive**	**False-Negative**	**True-Negative**	**False-Positive**
% patients	11.73%	5.27%	81.68%	1.32%
Mean LoS	5.33 hours	6.63 hours	6.63 hours	5.33 hours
**Mean ED LoS: SoC**	**6.46 hours**			
**Reduction in ED LoS with 1-h algorithm compared to SoC: 2.12 hours (33% reduction from SoC)**				

Abbreviations: h, hour; ED LoS, emergency department length of stay; SoC, standard of care

Table 5 presents the estimated costs (UK, Germany, and Switzerland perspective) associated with using SoC or the 1-h algorithm for the diagnosis of AMI. The costs are detailed for patients with true-positive, false-negative, true-negative, and false-positive diagnoses using the 1-h algorithm, including “rule-in,” “rule-out,” and “observation” patients, and SoC. The 1-h algorithm was associated with cost savings of £2,081 (46% reduction) per patient compared to SoC. The mean cost savings in the ED varied by study site, with Barcelona, Milan, Sydney, and Heidelberg showing higher cost savings per patient (range: £3,480 to £7,089), and Padova, Stockholm, Baltimore, and Detroit showing a lower reduction in cost savings per patient (range: £481 to £1,329; Fig. I in S1 Appendix). Using the unit costs from the Germany and Switzerland national payer perspectives resulted in cost savings of 38%, and 40%, respectively, with 1-h algorithm compared to the SoC (Table 5).

**Table 5:** Cost consequences 1-h algorithm and SoC.

	UK				Germany				Switzerland			
	True-Positive	False-Negative	True-Negative	False-Positive	True-Positive	False-Negative	True-Negative	False-Positive	True-Positive	False-Negative	True-Negative	False-Positive
**1-h Algorithm**												
Share of cost by diagnosis result*	**£634**	**£67**	**£1,624**	**£154**	**€ 582**	**€ 86**	**€ 670**	**€ 166**	**CHF 2,392**	**CHF 426**	**CHF 5,162**	**CHF 689**
**Rule-in/rule-out**	**£452**	**£16**	**£1,412**	**£132**	**€ 420**	**€ 26**	**€ 419**	**€ 145**	**CHF 1,813**	**CHF 125**	**CHF 4,160**	**CHF 609**
Before ED admission	£5	£0	£5	£1	€ 6	€ 0	€ 1	€ 2	CHF 13	CHF 0	CHF 3	CHF 3
During ED stay	£218	£12	£1,219	£55	€ 92	€ 1	€ 99	€ 23	CHF 601	CHF 28	CHF 2,756	CHF 153
Post ED discharge	£229	£0	£32	£58	€ 322	€ 0	€ 13	€ 82	CHF 1,198	CHF 1	CHF 72	CHF 304
Cost of alternative diagnosis	£0	£3	£156	£17	€ 0	€ 25	€ 306	€ 38	CHF 0	CHF 96	CHF 1,329	CHF 149
**Observation**	**£182**	**£52**	**£212**	**£22**	**€ 162**	**€ 60**	**€ 251**	**€ 22**	**CHF 579**	**CHF 301**	**CHF 1,002**	**CHF 79**
Before ED admission	£0	£0	£2	£0	€ 0	€ 0	€ 1	€ 0	CHF 1	CHF 0	CHF 4	CHF 0
During ED stay	£85	£39	£60	£10	€ 28	€ 4	€ 20	€ 3	CHF 211	CHF 90	CHF 142	CHF 24
Post ED discharge	£97	£7	£104	£11	€ 134	€ 9	€ 139	€ 15	CHF 368	CHF 30	CHF 462	CHF 41
Cost of alternative diagnosis	£0	£6	£46	£2	€ 0	€ 47	€ 91	€ 3	CHF 0	CHF 181	CHF 393	CHF 14
**Total cost per patient**	**£2,480**				**€ 1,504**				**CHF 8,668**			
**SoC**												
**Costs**	**£823**	**£232**	**£3,406**	**£100**	**€ 733**	**€ 269**	**€ 1,312**	**€ 98**	**CHF 2,614**	**CHF 1,354**	**CHF 10,151**	**CHF 356**
Before ED admission	£5	£1	£9	£1	€ 6	€ 0	€ 4	€ 1	CHF 13	CHF 1	CHF 16	CHF 1
During ED stay	£384	£176	£2,723	£43	€ 125	€ 18	€ 275	€ 14	CHF 947	CHF 405	CHF 6,285	CHF 107
Post ED discharge	£434	£30	£467	£49	€ 602	€ 40	€ 625	€ 68	CHF 1,654	CHF 134	CHF 2,080	CHF 186
Cost of alternative diagnosis	£0	£26	£208	£7	€ 0	€ 211	€ 407	€ 16	CHF 0	CHF 814	CHF 1,770	CHF 62
**Total cost per patient**	**£4,561**				**€ 2,412**				**CHF 14,475**			
**Cost savings: 1-h algorithm compared to SoC**	**£2,081 (46% savings)**				**€ 908 (38% savings)**				**CHF 5,807 (40% savings)**			

Abbreviations: CHF, Swiss franc; ED, emergency department; h, hour; SoC, standard of care; UK, United Kingdom; Currencies are reported in 2016 Great British Pounds (£), 2016 Euros (€), and 2016 Swiss Francs (CHF)

*Proportion of patients with diagnosis result multiplied by cost of managing per patient

### Sensitivity analysis

One-way sensitivity analysis indicated that the coefficients for study site, chest pain, dyspnea, congestive heart failure, used in the multivariate regression model for LoS of patients with AMI and non-AMI diagnosis influenced the reduction in LoS for the 1-h algorithm compared to SoC (Fig. J in S1 Appendix). Similarly, the cost savings with the 1-h algorithm compared to SoC were influenced by the coefficients for study site, chest pain, dyspnea, congestive heart failure, and were used in the multivariate regression model for LoS and sensitivity estimates for SoC (Fig. K in S1 Appendix).

## Discussion

Although the clinical performance of the 1-h algorithm has been previously studied in the four robust diagnostic studies using central adjudication,[16, 17] there is limited evidence on the economic outcomes of 1-h algorithm and its value compares with that of the current SoC in the ED setting. To help address this information gap, our study compared 1-h algorithm and SoC based on four outcomes–diagnostic accuracy, LoS, RU, and cost consequences.

Key findings of our analysis included that 1-h algorithm was estimated to have higher sensitivity and slightly lower specificity compared to SoC. An improvement in sensitivity with the 1-h algorithm was associated with a higher proportion of true-positives, allowing a more accurate AMI diagnosis compared to SoC. The higher sensitivity on the 1-h algorithm may be explained by the use of hs-cTnT, which allows the detection of lower limits of cTnT concentration.[15] The lower specificity of the 1-h algorithm compared to SoC was associated with a higher proportion of false-positives, a group who receive unnecessary follow-up of AMI. The lower detection limits of the high-sensitivity assay tend to pick up mild elevations in troponin markers caused by alternative underlying conditions;[16] and this may explain the slightly higher proportion of false-positives with the use of 1-h algorithm compared to SoC. Early coronary angiography may be considered as the appropriate consequence of early “rule-in” by the 1-h algorithm in the vast majority of patients. Of note, most patients “ruled-in” with diagnoses other than AMI have unstable angina, myocarditis, heart failure, or Takotsubo cardiomyopathy, all of which require coronary angiography for diagnosis and/or treatment. This is of particular relevance given that a recent paper showed that the increased sensitivity of hs-cTnT does not impact the rate of angiography, so suggesting that the current difference between assays in terms of specificity may not be statistically significant or economically consequential.[29]

The performance of the 1-h algorithm can be validated with previously published studies. The specificity of “rule-in” zone is 95% and the sensitivity of the “rule-out” zone in our analysis was 95%, and these results are similar to the findings of the APACE trial.[16, 17] The algorithm’s diagnostic accuracy is based on the weighted performance of “rule-in”/”rule-out” and “observation” zone. Due to the absence of data on the “observation” zone and to be conservative, we have assumed the performance to be similar to the SoC. In previously published economic analyses, the sensitivity and specificity of SoC has been assumed to be 100%;[32] however, our analysis estimated a lower sensitivity of 69%. This may be because our definition for SoC was based on the working diagnosis at the time of ED discharge and not a confirmed diagnosis at the end of hospital discharge.

Our analysis found that the 1-h algorithm is associated with a reduction in ED LoS of 2.1 hours (33%) compared to the SoC. Faster “rule-in” may enable AMI patients to receive treatment sooner and avoid long-term complications and mortality due to delayed diagnosis.[4, 5] On the other hand, faster “rule-out” may allow ED physicians to focus on pursuing alternative diagnoses. For all patients, the shorter length of stay will help with allocating ED facility and personnel time to other urgent care patients.

The multivariate regression model showed that both clinical characteristics and study sites are significant predictors of ED LoS for SoC patients. The ED LoS is dependent on organizational and administrative considerations. These include the fact that individual countries have to follow different guidelines and administrative constraints, and sites are unique in how they manage emergency care, with there being large variation in resource availability, staff availability and laboratory resources. For example, patients may get cardiac care in the emergency department due to resource limitations. Since we could not account for specific organizational and administrative constraints, including study site as predictors was considered as the next best alternative.

The algorithm’s LoS was the weighted average for rule-in, rule-out, and observation zone. Patients categorized as rule-in and rule-out were estimated to have greater reduction in LoS (2.6 hours; 41%) compared to the observation zone (assumed to be same as SoC). The primary drivers for the LoS for rule-in and rule-out were the time to second blood draw and the time between the second blood draw and ED discharge. The TRAPID-AMI study observed that the time to second blood draw was around 1.7 hours with no marked site-specific variability. However, the time between the second blood draw and ED discharge was dependent on laboratory turnaround time and ED processing time for discharge. Since the current input of 1.0 hour was based on expert opinion, we tested between 1 and 3 hours, and under all scenarios, the 1-h algorithm was associated with reduction in LoS compared to the SoC.

With the 1-h algorithm using the hs-cTnT assay, most patients are classified as “rule-in” or “rule-out” after two blood draws, therefore, leading to reductions in the use of resources in the ED. Additional blood draws, ECGs, imaging studies, and personnel time used to investigate the likeliness of acute AMI will not be required. However, this finding is based on the assumption that the 1-hour algorithm will be applied absolutely. The extent to which it has been implemented in clinical practice is not yet known and its actual impact on RU can only be validated in the future. It is also currently unclear whether clinicians in the ED will wait for additional blood draws, or ECGs, before they can provide a definitive diagnosis.

Furthermore, RU for alternative diagnosis may vary depending on underlying patient condition. For example, patients suspected of pulmonary embolism or aortic dissection require a d-dimer test and CT scan for definitive diagnosis.[14] In the TRAPID-AMI study, the proportion of patients with pulmonary embolism and aortic dissection was < 1%, which may have caused us to underestimate RU for “rule-out” patients. Alternative diagnosis identified in the TRAPID-AMI study included unstable angina, hypertensive crisis, arrhythmia, gastrointestinal disorder, musculoskeletal disorder, and anxiety syndrome. Based on expert opinion, these diagnoses are not expected to require additional RU compared to current requirements of 1-h algorithm.

The 1-h algorithm led to reductions in costs compared to the SoC (£2,081; 46%), and may translate to the cost savings on a national population level. For example, a total of 11,538,268 ED visits occur in a year in the UK,[33] of which 576,913 patients present to the ED with suspected AMI in a year (with 5% of ED visits relating to suspected of AMI[1]); therefore, this would lead to a reduction in costs for approximately 575,000 patients. The primary drivers for the reduction in costs is the expected reduction in RU. Based on expert opinion, both “rule-in” and “rule-out” patients require two blood draws. Costs associated with serial blood sampling, ECGs, and imaging tests are avoided by the use of the algorithm compared to SoC. The resource-use reduction and associated cost savings is specific to the ED setting and hospital facility. Furthermore, the resources such as ED bed space and staff time can be efficiently used for other patients. The use of 1-h algorithm may result in patients requiring medical resources in other outpatient setting, such as outpatient stress test for rule-out categories; hence, the cost savings should be seen in the scope of emergency department and may not accurately present the impact in the societal sense.

From the previously published studies,[19–23] Kaambwa[22] and Vaidya[23] presented the results of cost-effectiveness analyses of the hs-cTnT assay compared with the standard cTn assay. Both studies reported that the hs-cTnT assay is associated with higher costs (by €31 or $1,285), higher quality-adjusted life-years (0.004), and more adverse events avoided (0.0120) per patient. Our study findings are aligned, generally, with Kaambwa and Vaidya regarding improvement in diagnostic accuracy (fewer clinical adverse events). However, based on the current analysis, the 1-h algorithm using the hs-cTnT assay results in cost savings, which is contradictory to results presented by the published studies. Both Kaambwa and Vaidya evaluated the use of the hs-cTnT assay at six-hour intervals; our study assesses the use of 1-h algorithm, which is an accelerated diagnostic protocol. Consequently, there are differences in diagnostic accuracy. Furthermore, both studies show that the diagnostic accuracy of the standard cTn assay is similar to the hs-cTnT assay; this observation was not validated in the TRAPID-AMI study (Table 1). Lastly, none of the previously published analyses consider the cost associated with a missed diagnosis of AMI (false negative), erroneous diagnosis of AMI (false positive), and the cost of alternative diagnosis (true negative). These differences in modeling methods and inputs may explain the contradictory results regarding incremental costs associated with the use of the 1-h algorithm compared with the standard cTn assay.

We estimate that the 1-h algorithm would reduce 2.1 hours of bed space per patient, which would result in savings of approximately 50,000 days of bed space per year, considering 573,916 cases per year of suspected AMI. Since RU varied as per the study site, the associated cost consequences showed a large variation across countries and organizations. Another driver for reduction in costs is the improved sensitivity with 1-h algorithm compared to the SoC. Since 1-h algorithm is associated with lower proportion of false-negatives, costs associated with misdiagnosis are avoided.

### Limitations

Our study presents a number of limitations. Firstly, the physicians were blinded to the results of the 1-h algorithm in the TRAPID-AMI[17] study, therefore, the ED LoS and associated resource use were mostly based on modeling assumptions and expert opinion. Further studies will be required to assess the impact of the implementation of the 1-h algorithm in practice, on time to diagnosis and length of stay, and RU and associated costs.

Another key limitation of our study was our hypothesis that patients put in the observation zone would be equivalent to SoC, as regards to diagnostic accuracy, length of stay, RU, and costs. This assumption was deemed conservative as it possibly penalizes the performance of the 1-h algorithm. Further work will be needed to determine the performance of the algorithm in this population.

The TRAPID-AMI[17] study was restricted to the use of hs-cTnT; therefore, the results may or may not generalizable to other troponin assays (such as cardiac troponin I). Their use in practice, coupled with the 1-h algorithm, might provide some insights as to the generalizability of our results.

The analysis of the TRAPID-AMI[17] data showed very large variation between investigative sites on all variables. Furthermore, there was variation across sites with regards to treatment practice for SoC, including diagnostic techniques (availability of hs-cTnT), and treatment patterns. As previously discussed, this is due to sample size, country-specific and site-specific guidelines, and administrative constraints. In addition, our cost results were based on Switzerland, Germany, and the UK, and resource funding in the ED and costs are highly setting-specific and hence costs results are not applicable to other countries and other settings of care (such as ambulatory care).

Finally, the TRAPID-AMI trial was conducted in centers with experience in applying the 2011 ESC guidelines on hs-cTnT, and generalizability of analysis results to other centers with less research activities may or may not be possible depending on management patterns followed, education, and validation concepts.

## Supporting information

S1 Appendix **Fig. A in S1 Appendix. Mean Length of Stay for Patients with AMI and non-AMI Diagnosis by Study Sites: SoC** Abbreviations: AMI, acute myocardial infarction; LoS, length of stay; SoC, standard of care. **Fig. B in S1 Appendix. Mean Time to 2**^**nd**^ **Blood Draw for Patients Receiving SoC by Study Sites** Abbreviations: ED, emergency department. **Fig. C in S1 Appendix. Mean Length of Stay for Rule-in, Rule-out, and Observation Categorization of 1-h Algorithm** Abbreviations: AMI, acute myocardial infarction; ED, emergency department; h, hour; LoS, length of stay; SoC, standard of care. **Fig. D in S1 Appendix. Mean Length of Stay for Rule-in, Rule-out, and Observation Categorization of 1-h Algorithm by Study Sites** Abbreviations: h, hour; LoS, length of stay *Weighted average of rule-in/ rule-out and observation (assumed same as SoC). **Fig. E in S1 Appendix. Impact of Time between Second Blood Draw and ED Discharge on Analysis Results. Fig. F1 in S1 Appendix. Mean Number of Blood Draws by AMI and non-AMI Diagnosis and Study Sites: SoC** Abbreviations: AMI, acute myocardial infarction; SoC, standard of care. **Fig. F2 in S1 Appendix. Mean Number of ECGs by AMI and non-AMI Diagnosis and Study Sites: SoC** Abbreviations: AMI, acute myocardial infarction; SoC, standard of care. **Fig. F3 in S1 Appendix. Proportion of Patients Receiving CT and MRI by Study Sites: SoC** Abbreviations: CT, computed tomography scan; MRI, magnetic resonance imaging. **Fig. G in S1 Appendix. Proportion of Patients Receiving Procedures by Study Sites: SoC** Abbreviations: PTCA, percutaneous transluminal coronary angiography. **Fig. H in S1 Appendix. Reduction in LoS for 1-h Algorithm Compared to SoC by Study Sites** Abbreviations: LoS, length of stay; SoC, standard of care *Padova and Stockholm were associated with an increase in LoS with 1-h algorithm compared to SoC. **Fig. I in S1 Appendix. Cost Savings with 1-h Algorithm Compared to SoC by Study Sites** Abbreviations: h, hour; SoC, standard of care. **Fig. J in S1 Appendix. Tornado Diagram: Reduction in LoS for 1-h Algorithm Compared to SoC–Overall Population** Abbreviations: AMI, acute myocardial infarction; ED, emergency department; LoS, length of stay; SE, standard error. **Fig. K in S1 Appendix. Tornado Diagram: Cost Savings with 1-h Algorithm Compared to SoC–Overall Population** Abbreviations: AMI, acute myocardial infarction; ED, emergency department; LoS, length of stay; SE, standard error. **Table A in S1 Appendix. Resource Use for Patients with AMI and non-AMI Diagnosis: SoC** Abbreviations: SoC, standard of care; ED, emergency department; N, number of patients; ECG, electrocardiogram; CT, computed tomography scan; MRI, magnetic resonance imaging; CABG, coronary artery bypass graft surgery; PTCA, percutaneous transluminal coronary angiography *Unit cost per hour applied for the mean LoS **Unit cost for electrocardiogram monitoring or stress testing. **Table B in S1 Appendix. Resource Use for Patients with Rule-in and Rule-out: 1-h Algorithm** Abbreviations: SoC, standard of care; ED, emergency department; N, number of patients; ECG, electrocardiogram; CT, computed tomography scan; MRI, magnetic resonance imaging; CABG, coronary artery bypass graft surgery; PTCA, percutaneous transluminal coronary angiography *Assumed same as SoC **Physician, nurse, and ED stay is considered in the cost analysis. **Table C in S1 Appendix. Additional Cost for Alternative Diagnosis per Patient** Abbreviations: AMI, acute myocardial infarction; CHF, Swiss franc; UK, United Kingdom *True-negative patients will accrue the cost of treating underlying condition **False-negative patients will accrue the cost of misdiagnosis and repeat inpatient visit for AMI ***False-positive patients will accrue the cost of AMI treatment associated with misdiagnosis.
(DOCX)

## Abbreviations

AMI: acute myocardial infarction
APACE: Advantageous Predictors of Acute Coronary Syndrome Evaluation Study
CABG: coronary artery bypass grafting
CEC: clinical endpoint committee
CHF: Swiss franc
CI: confidence interval
CT: computed tomography
cTn: cardiac troponin
ECG: electrocardiogram
ED: emergency department
h: hour
hs-cTn: high-sensitivity cardiac troponin
LoS: length of stay
MRI: magnetic resonance imaging
NPV: negative predictive value
PPV: positive predictive value
PTCA: percutaneous transluminal coronary angiography
RU: resource utilization
SE: standard error
SoC: standard of care
TRAPID-AMI: The High Sensitivity Cardiac Troponin T Assay for Rapid Rule-out of Acute Myocardial Infarction
UK: United Kingdom
