# Correction: Economic evaluation of the one-hour rule-out and rule-in algorithm for acute myocardial infarction using the high-sensitivity cardiac troponin T assay in the emergency department

**DOI:** 10.1371/journal.pone.0191348

**Published:** 2018-01-11

**Authors:** Apoorva Ambavane, Bertil Lindahl, Evangelos Giannitsis, Julie Roiz, Joan Mendivil, Lutz Frankenstein, Richard Body, Michael Christ, Roland Bingisser, Aitor Alquezar, Christian Mueller, for the TRAPID-AMI investigators

The third author’s name is spelled incorrectly. The correct name is: Evangelos Giannitsis.
