# Supplementary material for: Economic evaluation of the one-hour rule-out and rule-in algorithm for acute myocardial infarction using the high-sensitivity cardiac troponin T assay in the emergency department
Source: PLoS One. 2017 Nov 9;12(11):e0187662. doi: 10.1371/journal.pone.0187662 (PMC5679593; doi:10.1371/journal.pone.0187662)
Supplement: S1 Appendix — Fig. A in S1 Appendix. Mean Length of Stay for Patients with AMI and non-AMI Diagnosis by Study Sites: SoC Abbreviations: AMI, acute myocardial infarction; LoS, length of stay; SoC, standard of care. Fig. B in S1 Appendix. Mean Time to 2nd Blood Draw for Patients Receiving SoC by Study Sites Abbreviations: ED, emergency department. Fig. C in S1 Appendix. Mean Length of Stay for Rule-in, Rule-out, and Observation Categorization of 1-h Algorithm Abbreviations: AMI, acute myocardial infarction; ED, emergency department; h, hour; LoS, length of stay; SoC, standard of care. Fig. D in S1 Appendix. Mean Length of Stay for Rule-in, Rule-out, and Observation Categorization of 1-h Algorithm by Study Sites Abbreviations: h, hour; LoS, length of stay *Weighted average of rule-in/ rule-out and observation (assumed same as SoC). Fig. E in S1 Appendix. Impact of Time between Second Blood Draw and ED Discharge on Analysis Results. Fig. F1 in S1 Appendix. Mean Number of Blood Draws by AMI and non-AMI Diagnosis and Study Sites: SoC Abbreviations: AMI, acute myocardial infarction; SoC, standard of care. Fig. F2 in S1 Appendix. Mean Number of ECGs by AMI and non-AMI Diagnosis and Study Sites: SoC Abbreviations: AMI, acute myocardial infarction; SoC, standard of care. Fig. F3 in S1 Appendix. Proportion of Patients Receiving CT and MRI by Study Sites: SoC Abbreviations: CT, computed tomography scan; MRI, magnetic resonance imaging. Fig. G in S1 Appendix. Proportion of Patients Receiving Procedures by Study Sites: SoC Abbreviations: PTCA, percutaneous transluminal coronary angiography. Fig. H in S1 Appendix. Reduction in LoS for 1-h Algorithm Compared to SoC by Study Sites Abbreviations: LoS, length of stay; SoC, standard of care *Padova and Stockholm were associated with an increase in LoS with 1-h algorithm compared to SoC. Fig. I in S1 Appendix. Cost Savings with 1-h Algorithm Compared to SoC by Study Sites Abbreviations: h, hour; SoC, standard of care. Fig. J in S1 Appendix. Tornado D [file pone.0187662.s001.docx]

Economic evaluation of the one-hour rule-out and rule-in algorithm for acute myocardial infarction using the high-sensitivity cardiac troponin T assay in the emergency department

Apoorva Ambavane^1*^, Bertil Lindahl^2^^, Evangelos Giannitis^3^^, Julie Roiz^1^, Joan Mendivil^4#^, Lutz Frankenstein^5^, Richard Body^6^^, Michael Christ^7^^, Roland Bingisser^8^, Aitor Alquezar^9^, Christian Mueller^10^^

^1^ Modeling and Simulation, Evidera, London, UK

^2^ Department of Medical Sciences, Uppsala University and Uppsala Clinical Research Center, Uppsala, Sweden

^3^ Medizinische Klinik III, University Heidelberg, Heidelberg, Germany

^4^ Previous employment: Market Access, Roche Diagnostics International Ltd., Rotkreuz, Switzerland

^5^ Department of Cardiology, Angiology, Pulmonology; University Hospital of Heidelberg, Heidelberg, Germany

^6^ Emergency Department, Central Manchester University Hospitals NHS Foundation Trust, Manchester, UK

^7^ Department of Emergency and Critical Care Medicine, Paracelsus Medical University, Nuremberg General Hospital, Nuremberg, Germany

^8^ Emergency Department, University of Basel, University Hospital, Basel, Switzerland

^9^ Servei de Urgencies. Hospital de Sant Pau, Barcelona, Spain

^10^ Department of Cardiology and Cardiovascular Research Institute Basel, University Hospital Basel, Basel, Switzerland

^*^ Corresponding Author

E-mail: apoorva.ambavane@evidera.com

^#^ Current employment: Global Health Economics, Outcomes and Epidemiology, Shire International GmbH, Zug, Switzerland

^^^ On behalf of the TRAPID-AMI investigators

# S1 Appendix

## Length of stay

**Fig. A in S1 Appendix. Mean Length of Stay for Patients with AMI and non-AMI Diagnosis by Study Sites: SoC**

*Overall: Mean LoS Non-AMI = 6.6 hours*

*Overall Mean LoS AMI = 5.3 hours*

Abbreviations: AMI, acute myocardial infarction; LoS, length of stay; SoC, standard of care

**Fig. B in S1 Appendix. Mean Time to 2^nd^ Blood Draw for Patients Receiving SoC by Study Sites**

*Weighted average of expert opinion/clinical experts*

Abbreviations: ED, emergency department

**Fig. C in S1 Appendix. Mean Length of Stay for Rule-in, Rule-out, and Observation Categorization of 1-h Algorithm**


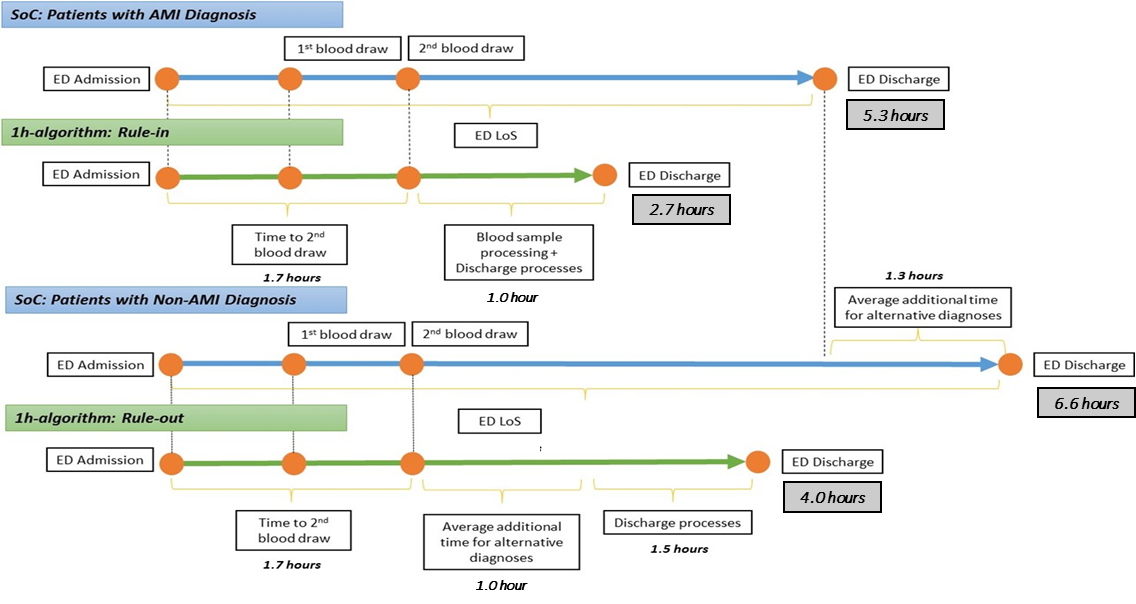


Abbreviations: AMI, acute myocardial infarction; ED, emergency department; h, hour; LoS, length of stay; SoC, standard of care

**Fig. D in S1 Appendix. Mean Length of Stay for Rule-in, Rule-out, and Observation Categorization of 1-h Algorithm by Study Sites**

*Overall: Mean LoS Rule-out = 4.0 hours*

*Overall Mean LoS Rule-in = 2.7 hours*

*Overall Mean LoS 1-h Algorithm = 4.3 hours**

Abbreviations: h, hour; LoS, length of stay

**Weighted average of rule-in/ rule-out and observation (assumed same as SoC)*

**Fig. E in S1 Appendix. Impact of Time between Second Blood Draw and ED Discharge on Analysis Results**

**
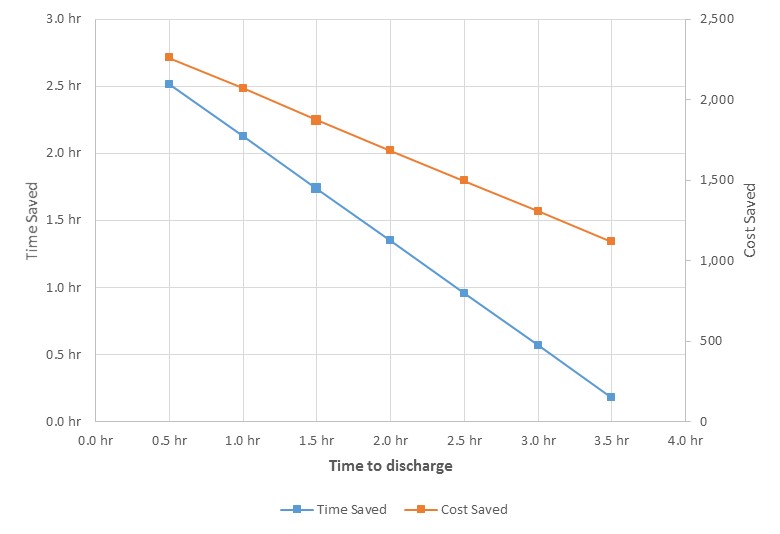
**

## Resource use

**Table A in S1 Appendix. Resource Use for Patients with AMI and non-AMI Diagnosis: SoC**

|  | **Unit Costs** | | | **% of Patients with AMI Treated During** | | | **% of Patients with Non-AMI Treated During** | | |
| --- | --- | --- | --- | --- | --- | --- | --- | --- | --- |
| **List of Tests** | **UK** | **Germany** | **Switzerland** | **Before ED Admission** | **During ED Admission** | **Post ED Admission** | **Before ED Admission** | **During ED Admission** | **Post ED Admission** |
| 1st blood draw | £5 | €11 | €23 | 0% | 99% | 1% | 1% | 99% | 0% |
| 2nd blood draw |  |  |  | 0% | 91% | 9% | 0% | 97% | 3% |
| 3rd blood draw |  |  |  | 0% | 66% | 23% | 0% | 82% | 10% |
| 4th blood draw |  |  |  | 0% | 35% | 57% | 0% | 44% | 40% |
| Initial ECG | £52** | €8 | €30 | 8% | 90% | 2% | 16% | 84% | 0% |
| 1st subsequent ECG |  |  |  | 0% | 36% | 31% | 0% | 31% | 17% |
| 2nd subsequent ECG |  |  |  | 0% | 8% | 32% | 0% | 5% | 12% |
| 3rd subsequent ECG |  |  |  | 0% | 3% | 21% | 0% | 1% | 4% |
| 4th subsequent ECG |  |  |  | 0% | 1% | 18% | 0% | 0% | 2% |
| 5th subsequent ECG |  |  |  | 0% | 1% | 12% | 0% | 0% | 1% |
| 6th subsequent ECG |  |  |  | 0% | 0% | 7% | 0% | 0% | 1% |
| 7th subsequent ECG |  |  |  | 0% | 0% | 5% | 0% | 0% | 0% |
| 8th subsequent ECG |  |  |  | 0% | 0% | 2% | 0% | 0% | 0% |
| 9th subsequent ECG |  |  |  | 0% | 0% | 1% | 0% | 0% | 0% |
| 10th subsequent ECG |  |  |  | 0% | 0% | 1% | 0% | 0% | 0% |
| CT Scan | £113 | €69 | €241 | 0% | 4% | 3% | 0% | 5% | 2% |
| MRI | £184 | €127 | €260 | 0% | 1% | 1% | 0% | 0% | 1% |
| ECHO | £57 | €28 | €346 | 0% | 7% | 54% | 0% | 6% | 17% |
| PTCA | £2,751 | €3,790 | €16,281 | 1% | 9% | 48% | 0% | 0% | 5% |
| Invasive Angiography | £162 | €327 | €562 | 1% | 12% | 67% | 0% | 1% | 14% |
| Stent | £2,752 | €3,790 | €249 | 1% | 9% | 46% | 0% | 0% | 4% |
| CABG | £8,154 | €13,125 | €50,624 | 0% | 0% | 5% | 0% | 0% | 0% |
| Physician consult* | £150 | €22 | €1,089 | 100% | 100% | 100% | 100% | 100% | 100% |
| Nurse consult* | £121 | €0 | €0 | 100% | 100% | 100% | 100% | 100% | 100% |
| ED Stay* | £214 | €14 | €25 | 100% | 100% | 100% | 100% | 100% | 100% |

Abbreviations: SoC, standard of care; ED, emergency department; N, number of patients; ECG, electrocardiogram; CT, computed tomography scan; MRI, magnetic resonance imaging; CABG, coronary artery bypass graft surgery; PTCA, percutaneous transluminal coronary angiography

*Unit cost per hour applied for the mean LoS

**Unit cost for electrocardiogram monitoring or stress testing

**Table B in S1 Appendix. Resource Use for Patients with Rule-in and Rule-out: 1-h Algorithm**

| **Rule-in** | | | | | |
| --- | --- | --- | --- | --- | --- |
| **Resource** | **% Patients: Before ED Admission*** | **Resource** | **% Patients: During ED Admission**** | **Resource** | **% Patients: Post ED Discharge*** |
| Initial ECG | 8% | Initial ECG | 90% | PTCA | 58% |
| Invasive Angiography | 1% | 1st blood draw | 99% | Invasive Angiography | 80% |
| PTCA | 1% | ECHO | 7% | Stent | 55% |
| Stent | 1% | 4th subsequent ECG | 1% | CABG | 5% |
|  |  | 2nd blood draw | 91% |  |  |
|  |  | 1st subsequent ECG | 36% |  |  |
|  |  | CT Scan | 4% |  |  |
| **Rule-out** | | | | | |
| **Resource** | **% Patients: Before ED Admission*** | **Resource** | **% Patients: During ED Admission**** | **Resource** | **% Patients: Post ED Discharge** |
| 1st blood draw | 1% | Initial ECG | 84% | Outpatient Stress Test | 100% |
| 1st subsequent ECG | 0% | 1st blood draw | 99% |  |  |
| Initial ECG | 16% | 2nd blood draw | 97% |  |  |
| 2nd subsequent ECG | 0% |  |  |  |  |
| ECHO | 0% |  |  |  |  |

Abbreviations: SoC, standard of care; ED, emergency department; N, number of patients; ECG, electrocardiogram; CT, computed tomography scan; MRI, magnetic resonance imaging; CABG, coronary artery bypass graft surgery; PTCA, percutaneous transluminal coronary angiography

*Assumed same as SoC

**Physician, nurse, and ED stay is considered in the cost analysis

Table C in S1 Appendix. Additional Cost for Alternative Diagnosis per Patient

|  | **Cost of Inpatient Treatment** | | | **Proportion of Patients** | | |
| --- | --- | --- | --- | --- | --- | --- |
|  | **UK** | **Germany** | **Switzerland** | **True Negative*** | **False Negative**** | **False Positive***** |
| Unstable angina | £ 648 | € 1,411 | CHF 5,506 | 15% | 23% | 79% |
| Hypertensive crisis | £ 644 | € 1,431 | CHF 6,210 | 3% | 6% | 5% |
| Arrhythmia | £ 903 | € 2,117 | CHF 9,740 | 5% | 29% | 0% |
| Gastrointestinal disorder | £ 711 | € 1,545 | CHF 8,183 | 7% | 3% | 0% |
| Musculoskeletal disorder | £ 880 | € 616 | CHF 2,742 | 0% | 3% | 0% |
| Pulmonary embolism | £ 1,507 | € 3,114 | CHF 7,930 | 0% | 0% | 0% |
| Aortic dissection | £ 1,652 | € 2,211 | CHF 11,873 | 0% | 0% | 0% |
| Anxiety syndrome | £ 839 | € 549 | CHF 1,849 | 5% | 0% | 0% |
| Otherᵻ | £ 0 | € 0 | CHF 0 | 65% | 36% | 16% |
| AMI | £ 1,383 | € 2,924 | CHF 10,685 |  | 100% |  |
| Total Cost of Treatment - UK | | | | £ 255 | £ 1,878 | £ 546 |
| Total Cost of Treatment - Germany | | | | € 499 | € 4,007 | € 1,189 |
| Total Cost of Treatment - Switzerland | | | | CHF 2,166 | CHF 15,448 | CHF 4,674 |

Abbreviations: AMI, acute myocardial infarction; CHF, Swiss franc; UK, United Kingdom

*True-negative patients will accrue the cost of treating underlying condition

**False-negative patients will accrue the cost of misdiagnosis and repeat inpatient visit for AMI

***False-positive patients will accrue the cost of AMI treatment associated with misdiagnosis and repeat inpatient visit for underlying condition

ᵻ Assumed to be zero

**Fig. F1 in S1 Appendix. Mean Number of Blood Draws by AMI and non-AMI Diagnosis and Study Sites: SoC**

Abbreviations: AMI, acute myocardial infarction; SoC, standard of care

**Fig. F2 in S1 Appendix. Mean Number of ECGs by AMI and non-AMI Diagnosis and Study Sites: SoC**

Abbreviations: AMI, acute myocardial infarction; SoC, standard of care

**Fig. F3 in S1 Appendix. Proportion of Patients Receiving CT and MRI by Study Sites: SoC**

Abbreviations: CT, computed tomography scan; MRI, magnetic resonance imaging

**Fig. G in S1 Appendix. Proportion of Patients Receiving Procedures by Study Sites: SoC**

Abbreviations: PTCA, percutaneous transluminal coronary angiography

## Cost-consequence analysis

**Fig. H in S1 Appendix. Reduction in LoS for 1-h Algorithm Compared to SoC by Study Sites**

*Overall Mean = 2.1 hours*

Abbreviations: LoS, length of stay; SoC, standard of care

*Padova and Stockholm were associated with an increase in LoS with 1-h algorithm compared to SoC

**Fig. I in S1 Appendix. Cost Savings with 1-h Algorithm Compared to SoC by Study Sites**

Abbreviations: h, hour; SoC, standard of care

**Fig. J in S1 Appendix. Tornado Diagram: Reduction in LoS for 1-h Algorithm Compared to SoC – Overall Population**

Abbreviations: AMI, acute myocardial infarction; ED, emergency department; LoS, length of stay; SE, standard error

**Fig. K in S1 Appendix. Tornado Diagram: Cost Savings with 1-h Algorithm Compared to SoC – Overall Population**

Abbreviations: AMI, acute myocardial infarction; ED, emergency department; LoS, length of stay; SE, standard error
